# Different Methods of Secondary Intraocular Lens Implantation

**DOI:** 10.1155/2023/9847067

**Published:** 2023-12-19

**Authors:** Matteo Forlini, Boris Malyugin, Ike Ahmed, Gabor Scharioth, Rodolfo Mastropasqua, Alessandro Mularoni

**Affiliations:** ^1^San Marino State Hospital, Cailungo, San Marino; ^2^Fyodorov Eye Microsurgery State Institution, Moscow, Russia; ^3^University of Toronto, Toronto, Canada; ^4^Aurelios Augenzentrum, Recklinghausen, Germany; ^5^University of Modena, Modena, Italy

In modern cataract surgery, “in-the-bag” IOL placement is the ideal standard of care in order to allow excellent refractive results and fast visual recovery [1].

The precise positioning of the lens is crucial to achieve therapeutic effect, especially in case of toric lenses used to correct astigmatism. In this scenario, 1° misalignment reduces astigmatic correction by nearly 3.3%, whereas 30° misalignment might not correct or might increase astigmatism [2].

In everyday practice, several conditions may result in zonular loss and inadequate capsular support, such as lens dislocation in the vitreous chamber, posttraumatic cataract surgery, pseudoexfoliation, and Marfan and Ehlers–Danlos syndromes [3].

In this special issue, the reader will be able to cope with various surgical approaches which could be adopted in case there is the need to identify an alternative intraocular area to place the IOL.

These approaches include IOL implantation within the anterior chamber (AC-IOL), IOL fixated to the iris (IF-IOL), and IOL fixated to the sclera (SF-IOL) [4].

In AC-IOL placement, haptics are positioned in the iridocorneal angle: this is a faster and less complicated technique when compared with sutured IOLs; however, its use has been limited due to significant issues such as large corneal incision, bullous keratopathy, and secondary glaucoma [5, 6].

Furthermore, fixation to the iris involves suturing the haptics of a 3-piece IOL to the peripheral iris and it is useful in case of displacement of IOLs previously located in the sulcus and in all cases where sparing the conjunctiva or filtering bleb is needed [7].

On the other hand, this technique has been associated to secondary glaucoma, iris chafing, pigment dispersion, central macular edema (CME), and pupil distortion [8].

Another alternative surgical approach involves the Artisan Aphakic IOL (Ophtec BV, Groningen, The Netherlands), which is an iris-claw IOL currently used in Europe whose main feature is having two “claws” on both sides which allow enclavation to the iris tissue [9].

This technique has a flat learning curve, short surgical time, and low incidence of postoperative complications. On the negative side, it also has a slow visual recovery due to high postoperative astigmatism which creates a discomforting period of low visual acuity [10].

Scleral-fixated intraocular lenses need to be anchored to the sclera by sutures or sutureless techniques: in both cases, the technique is more complex than AC-IOL or IF-IOL and an anterior or pars plana vitrectomy is required as well as an anterior chamber maintainer in order to preserve intraocular pressure during surgery [11].

Scleral fixation allows IOL placement in the posterior chamber leading to greater refractive results and it is useful in case of a low endothelial cell count. On the other hand, this technique might face suture-related complications, such as breakage and conjunctival erosion, which are associated with a higher risk of endophthalmitis [12].

In recent years, new materials have been successfully introduced, such as Gore-Tex monofilament, which has superior tensile strength and increased durability compared to the previously used Prolene. This allows fixation of several nonfoldable scleral IOLs (such as Alcon CZ70BD and Bausch and Lomb Akreos A60) and thus reducing sutures-related complications [13].

Moreover, sutureless scleral fixation has become increasingly popular due to the absence of complications associated with large wounds or stitches [14].

First described by Shin Yamane in 2017, the flanged intrascleral IOL fixation technique is a double-needle technique which entails the externalization of two haptics using a 30-gauge thin-wall needle at 2 mm away from the limbus. After externalization, low-temperature cautery is performed at the tip of the haptics, creating flanges which can be embedded within the sclera [15].

It has also been proved that this technique can be safely performed with 27-gauge needles, extending its accessibility to countries where 30-gauge needles are not available [15].

In order to optimize refractive results, studies have underlined the urge to use the Yamane stabilizer, which allows the placement of 2 opposite sclerotomies at exactly 180°, and to heat the last 2 mm of the IOL haptics.

However, this technique has been standardized only with the preloaded 3-piece IOL (Kowa PU6AS, Japan), so further investigations regarding other types of IOLs are therefore needed [16].

In recent years, Carlevale et al. have developed a new foldable scleral IOL (Soleko) provided by scleral harpoons which enable sutureless anchorage to the sclera by a 23-gauge sclerotomy [10, 17].

The anchors allow precise centration, which was demonstrated by a vertical and horizontal tilting not exceeding 5°, and prevent posterior dislocation which might explain the low incidence of vitreous hemorrhage and retinal tears or detachment [2].

This special issue will also focus on expected refractive results of each of these techniques.

In fact, iris-claw IOL [18], flanged transscleral-fixated IOL (Yamane technique), and sutureless transscleral hook IOL fixation (Carlevale IOL) showed a similar functional recovery and a similar myopic shift.

At the moment, there is still no consensus on the target of spheric equivalent [11].

In conclusion, this special issue has a platter of original research articles and experimental studies, as well as case series on secondary intraocular lens implantation, illustrating and discussing refractive outcomes and how to deal with postoperative complications.

This work will hopefully offer readers a new perspective in dealing with insufficient capsular support and thus stimulating further research.

*Matteo Forlini* *Boris Malyugin* *Ike Ahmed* *Gabor Scharioth* *Rodolfo Mastropasqua* *Alessandro Mularoni*
